# Kidney Failure Associates With T Cell Exhaustion and Imbalanced Follicular Helper T Cells

**DOI:** 10.3389/fimmu.2020.583702

**Published:** 2020-09-29

**Authors:** Susan Hartzell, Sofia Bin, Chiara Cantarelli, Meredith Haverly, Joaquin Manrique, Andrea Angeletti, Gaetano La Manna, Barbara Murphy, Weijia Zhang, Josh Levitsky, Lorenzo Gallon, Samuel Mon-Wei Yu, Paolo Cravedi

**Affiliations:** ^1^Division of Nephrology, Department of Medicine, Icahn School of Medicine at Mount Sinai, New York, NY, United States; ^2^UO Nefrologia, Azienda Ospedaliera-Universitaria di Parma, Parma, Italy; ^3^Nephrology Service, Complejo Hospitalario de Navarra, Pamplona, Spain; ^4^Division of Nephrology, Dialysis, Transplantation, Istituto di Ricovero e Cura a Carattere Scientifico (IRCCS) Giannina Gaslini, Genoa, Italy; ^5^Department of Experimental Diagnostic and Specialty Medicine, University of Bologna Sant'Orsola- Malpighi Hospital, Bologna, Italy; ^6^Division of Gastroenterology, Department of Medicine, Feinberg School of Medicine, Northwestern University, Chicago, IL, United States; ^7^Division of Nephrology, Department of Medicine, Feinberg School of Medicine, Northwestern University, Chicago, IL, United States

**Keywords:** exhaustion, ESKD, dialysis (ESKD), T cell, treg, immune phenotype

## Abstract

Individuals with kidney failure are at increased risk of cardiovascular events, as well as infections and malignancies, but the associated immunological abnormalities are unclear. We hypothesized that the uremic milieu triggers a chronic inflammatory state that, while accelerating atherosclerosis, promotes T cell exhaustion, impairing effective clearance of pathogens and tumor cells. Clinical and demographic data were collected from 78 patients with chronic kidney disease (CKD) (*n* = 42) or end-stage kidney disease (ESKD) (*n* = 36) and from 18 healthy controls (HC). Serum cytokines were analyzed by Luminex. Immunophenotype of T cells was performed by flow cytometry on peripheral blood mononuclear cells. ESKD patients had significantly higher serum levels of IFN-γ, TNF-α, sCD40L, GM-CSF, IL-4, IL-8, MCP-1, and MIP-1β than CKD and HC. After mitogen stimulation, both CD4^+^ and CD8^+^ T cells in ESKD group demonstrated a pro-inflammatory phenotype with increased IFN-γ and TNF-α, whereas both CKD and ESKD patients had higher IL-2 levels. CKD and ESKD were associated with increased frequency of exhausted CD4^+^ T cells (CD4^+^KLRG1^+^PD1^+^CD57^−^) and CD8^+^ T cells (CD8^+^KLRG1^+^PD1^+^CD57^−^), as well as anergic CD4^+^ T cells (CD4^+^KLRG1^−^PD1^+^CD57^−^) and CD8^+^ T cells (CD8^+^KLRG1^−^PD1^+^CD57^−^). Although total percentage of follicular helper T cell (T_FH_) was similar amongst groups, ESKD had reduced frequency of T_FH1_ (CCR6^−^CXCR3^+^CXCR5^+^PD1^+^CD4^+^CD8^−^), but increased T_FH2_ (CCR6^−^CXCR3^−^CXCR5^+^PD1^+^CD4^+^CD8^−^), and plasmablasts (CD3^−^CD56^−^CD19^+^CD27^high^CD38^high^CD138^−^). In conclusion, kidney failure is associated with pro-inflammatory markers, exhausted T cell phenotype, and upregulated T_FH2_, especially in ESKD. These immunological changes may account, at least in part, for the increased cardiovascular risk in these patients and their susceptibility to infections and malignancies.

## Introduction

Despite significant improved survival in patients with chronic kidney disease (CKD) and end-stage kidney disease (ESKD) in the past decades, the all-cause mortality among these patients has remained exceedingly higher when compared to general population (1, 2). In particular, cardiovascular diseases, infections, and malignancies represent the leading causes of the mortality independent of concomitant comorbidities, such as diabetes or hypertension, suggesting a unique and systemic pathobiology in kidney failure (3–5).

In response to pathogen exposure or tissue damage, the body generates physiological and necessary inflammation to restore homeostasis. However, prolonged inflammatory signals alter T cell functions, leading to a state of exhaustion (6). Exhausted T cells, both CD4^+^ and CD8^+^, are characterized by the loss of their effector functions and the expression of inhibitory receptors such as PD-1 and KLRG1. Co-expression of these inhibitory receptors can be of additive effects (6). Functionally, exhausted T cells have impaired cytokine production upon stimulation, but retain some cytotoxicity and degranulation capability (7). Exhausted T cells might be reversed back to fully functional T cells; however, these functional changes become irreversible if stimulus such as inflammation sustains, and eventually lead to cell death (6, 8). Clinically, T cell exhaustion has been associated with unresolved infection and drug-resistant tumors, and emerging immunotherapy such as anti-PD-1 was designed to reverse functional deficits of exhausted T cells (7, 9). In addition, a single-cell analysis also suggested the existence of T cell exhaustion in the development of atherosclerosis (10). Therefore, it is possible that significantly increased risks of cardiovascular death, infection, and malignancy in kidney failure are mediated by exhausted T cells, as a markedly increased T cell exhaustion was seen in a pediatric CKD cohort (11). Follicular helper T cells (T_FH_), a subset of CD4^+^ T cells expressing CXC-chemokine receptor 5 (CXCR5), was initially discovered in the germinal centers (GC) of human tonsillar tissues (12). To produce high affinity antibodies against pathogens, B cell requires the assistance from T_FH_ to complete its maturation and class switch recombination inside the GC. Work related to T_FH_ in the kidneys has largely focused on kidney transplantations, and upregulated T_FH_ has been implicated in less favorable outcomes especially due to increased humoral rejections (13). On the other hand, T_FH_ are also linked to atherosclerosis, autoimmune diseases and some lymphoid tumors, suggesting their potential pathogenic role in promoting chronic inflammation (12, 14).

To date, data on T cell exhaustion or T_FH_ in adult CKD and ESKD remains scarce and incomplete. We hypothesized that an association between impaired renal function and exhausted T cell as well as follicular helper T cells, might explain the increased risk of infection, cancer, and cardiovascular disease among CKD and ESKD patients (15–20), and performed a comprehensive immunophenotyping on T cells.

## Materials and Methods

### Study Population

We included 42 CKD patients (stage 1–5 or with proteinuria) seen at Complejo Hospitalario de Navarra, Pamplona, Spain (Biobank Navarrabiomed; *n* = 32), or S. Orsola-Malpighi Hospital, University of Bologna, Bologna, Italy (*n* = 10), 36 ESKD patients who received hemodialysis for > 3 months at the Mount Sinai Kidney Center (*n* = 21), Northwestern Hospital in Chicago (*n* = 13), or Complejo Hospitalario de Navarra, Pamplona, Spain (Biobank Navarrabiomed; *n* = 2), and 18 age-matched healthy controls (HC). All individuals were enrolled from November 2017 to December 2019 (Table 1). Exclusion criteria were pregnancy, recent (<3 months) infectious episode requiring hospitalization, history of kidney transplant, use of immunosuppressive medications at the time of enrollment, inability to give consent, active malignancy. Samples and data from patients included in this study were processed following standard operating procedures with the appropriate approval of the Ethics and Scientific Committees of the participating centers.

**Table 1:** Patients' characteristics.

	**Healthy controls (*n* = 18)**	**CKD (*n* = 42)**	**ESKD (*n* = 36)**	***P***
Age (yr)	57.0 ± 8.4	56.1 ± 17.9	55.0 ± 14.1	0.64
Sex, *n* (%)	^◦^			0.26
Male	5 (41.7)	22 (52.4)	20 (55.6)	
Female	7 (58.3)	20 (47.6)	16 (44.4)	
Primary nephropathy, *n* (%)	na			0.18
ADPKD		14 (33.3)	3 (8.3)	
Diabetes mellitus		9 (21.4)	10 (27.8)	
Hypertension		8 (19.0)	11 (30.6)	
IgA		7 (16.7)	0	
FSGS		2 (4.8)	1 (2.8)	
MN		0	2 (5.6)	
Unspecified CKD		2 (4.8)	9 (25.0)	
CKD Stage	na		na	
1		2 (4.8)		
2		2 (4.8)		
3		4 (9.5)		
4		18 (42.9)		
5		16 (38.1)		
Dialysis vintage (yr)	na	na	4.5 ± 4.0	
Laboratory				
Serum creatinine (mg/dL)	na	3.4 ± 1.5	8.1 ± 3.9	0.0001
Proteinuria (g/24 h)	na	1.0 ± 1.5	na	
Serum albumin (g/dL)	na	4.0 ± 0.6	3.4 ± 0.5^*^	0.0003
Lymphocyte (#/μL)	na	1.5 ± 0.5	1.4 ± 0.7	0.5939
CRP (mg/dL)	na	na	26.0 ± 43^*^	
Ferritin (ug/L)	na	na	543.5 ± 801.1^*^	

*Data are average ± SD or n (%). ADPKD, autosomal dominant polycystic kidney disease; CKD, chronic kidney disease; MN, membranous nephropathy; CRP, C reactive protein; FSGS, focal segmental glomerulosclerosis; HD, hemodialyzed; IgA, IgA nephropathy; na, not available*;

◦ *data not available for 6 healthy controls*;

* *data not available for 13 ESKD patients. CKD stages were classified as follows: 1- eGFR > 90 with proteinuria; 2- eGFR 60–80 with proteinuria; 3- eGFR 30–60; 4- eGFR 15–30; 5- eGFR < 15 mL/min*.

### Luminex

We quantified plasma levels of CD40L, G-CSF/CSF-3, GM-CSF, IFN-γ, IL-1β, IL-10, IL-12p70, IL-13, IL-17A, IL-2, IL-4, IL-5, IL-6, IL-7, IL-8, MCP-1, MIP-1β, TNF-α, and TNF-β by Luminex® (xMAP® Technology). We created a multiplex panel combining two commercially available simplex kits: Human Custom Procarta Plex-19 plex (Invitrogen by Thermo Fisher Scientific, Cat. No. PPX-19-MXRWE2G) and Human High Sensitivity T Cell (Merck Millipore, Cat. No. HSTCMAG-28SK). The methodological details including assay protocol, standards and sensitivity are available at the manufacturer's website (http://www.thermofisher.com and http://www.merckmillipore.com, respectively). All samples were measured undiluted and in duplicates. The chemo/cytokine standards were assayed in the same way as patient samples. The data were collected using a xPONENT® software (Luminex, Austin, TX, USA).

### Flow Cytometry Analysis

PBMC were isolated from peripheral blood by Ficoll gradient and frozen for batched analysis. We designed six multicolor flow cytometry panels to quantify 60 T cell subsets along with two B cell subsets, and calculated the CD4^+^/CD8^+^ T cell ratio (Supplementary Table 1). The following fluorochrome-conjugated anti-human antibodies were used from BD Biosciences (San Jose, CA): CD3-FITC, CD3-PerCP-Cy5.5, CD4-APC, CD4-APC-Cy7, CD8-BV510, CD45RO-FITC, CD45RA-APC, CD45RA-APC-Cy7, CCR4-PE, CD27-PE, CD28-BV421, CD138-BV421, CCR6-BV421, CXCR3-PE, CCR7-A700, IL-17-BV786, IFN-γ-PE-Cy7, iso IgG1k-FITC, iso IgG1k-PE-Cy7, iso IgG2bk-APC, iso IgG1k-APC-Cy7, and iso IgG1k-BV510; from Biolegend (San Diego, CA): CD127-FITC, CD27-APC, CD57-PerCp-Cy5.5, CD19-BV510, PD-1-APC-Cy7, CXCR5-FITC, and TNFα-FITC; from eBioscience (San Diego, CA): CD4-PE-Cy7, and IL-2-PE; from Miltenyi Biotec (San Diego, CA): CD25-APC and KLRG1-PE; from Beckman Coulter (Brea, CA): CD38-PE-Cy7.

### Intracellular Cytokine Staining

We performed intracellular staining for IL-17, IL-2, IFN-γ, and TNF-α with extracellular markers for CD4^+^ and CD8^+^ after 5 h stimulation by mixture of PMA (Fisher, Waltham, MA), ionomycin (Fisher) and Golgi Plug (Millipore Sigma, Burlington, MA) at 37°C. We fixed and permeabilized the cells using Intracellular Fixation and Permeabilization Buffer Set (eBioscience), according to the manufacturer's instructions. Data were acquired (> 1 × 10^6^ events) on a 3-laser FACSLyric flow cytometer (BD Biosciences) and analyzed with FlowJo® software. Intracellular cytokines were recorded as percentages of CD4^+^ and CD8^+^ T cells.

### Statistical Analyses

Results were presented as mean and standard deviation or standard error unless stated otherwise. Comparison of continuous variables between groups was performed by Kruskal–Wallis test, unpaired *t*-test, and categorical variables by two-sided chi-square, where applicable. *P* < 0.05 was considered as statistically significant. No correction was made for multiple testing. Statistical analysis was performed using GraphpadPrism® version 8.4.2 software package (Graphpad Software Inc., San Diego, CA).

## Results

### Patients

Patient characteristics are presented in Table 1. The overall age of our cohort was 57.4 ± 15.7 years with no difference among the groups (57.0 ± 8.4 vs. 56.1 ± 17.9 vs. 55.0 ± 14.1 years for HC, CKD, and ESKD, respectively, *p* = 0.64). In CKD cohort, the most common cause of CKD was autosomal-dominant polycystic kidney disease (ADPKD) (*n* = 14, 33.3%), followed by diabetic kidney disease (DKD) (*n* = 9, 21.4%), hypertension (*n* = 8, 19.0%), IgA nephropathy (*n* = 7, 16.7%), focal segmental glomerulosclerosis (FSGS) (*n* = 2, 4.8%), and unspecified CKD (*n* = 2, 4.8%). In ESKD cohort, the most common cause of ESKD was hypertension (*n* = 11, 30.6%), followed by DKD (*n* = 10, 27.8%), unspecified CKD (*n* = 9, 25.0%), ADPKD (*n* = 3, 8.3%), membranous nephropathy (MN) (*n* = 2, 5.6%), and FSGS (*n* = 1, 2.8%).

### Serum Cytokines

**Figure 1:**
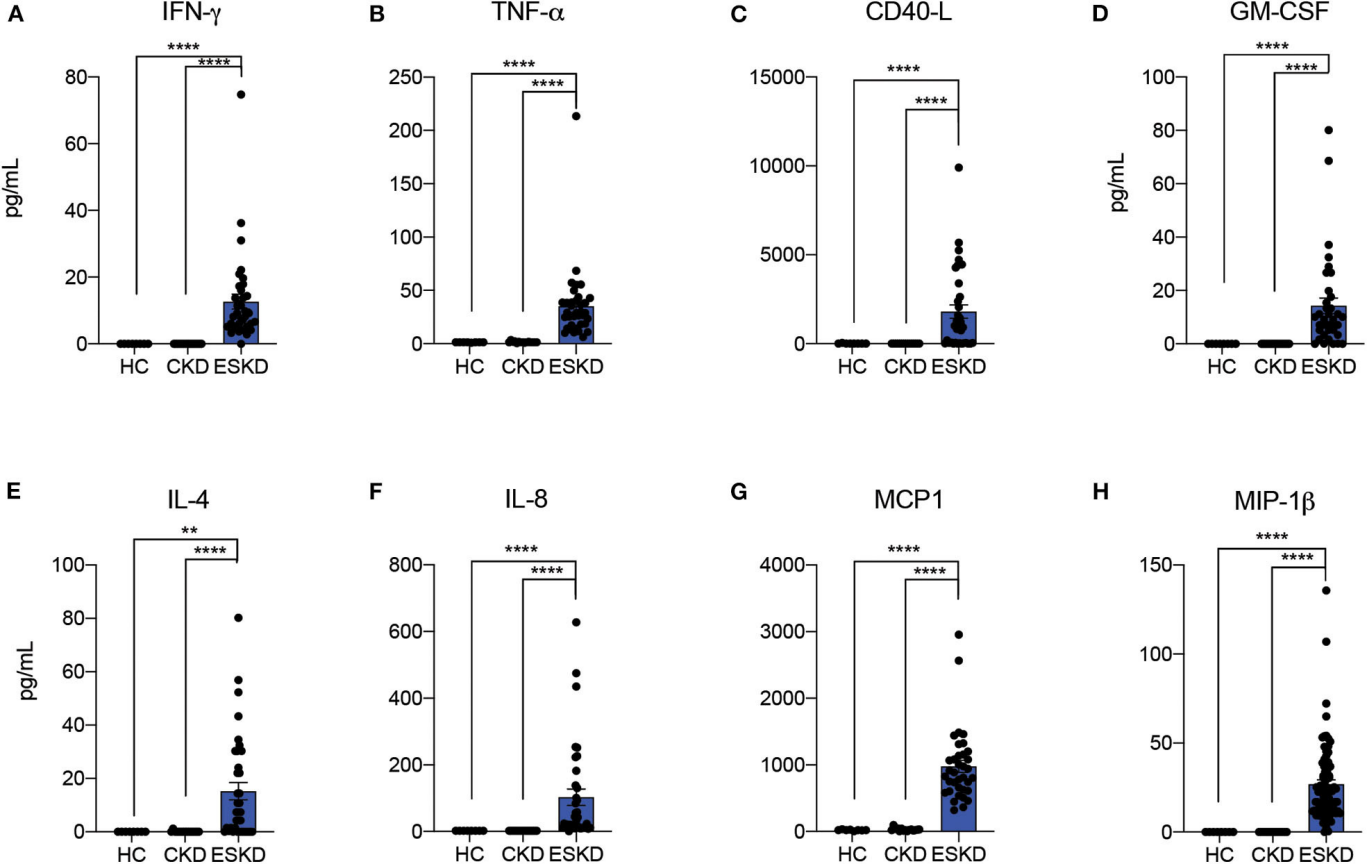
Serum cytokine analysis by Luminex. Data are represented as mean and standard error of the mean (SEM). Each dot represents an individual value. **P < 0.01; ****P < 0.0001. Kruskal–Wallis test.

To start testing the inflammatory status of patients with kidney failure, we measured inflammatory cytokines: soluble CD40 ligand (sCD40-L), granulocyte-macrophage colony-stimulating factor (GM-CSF), granulocyte colony-stimulating factor (G-CSF), interferon-gamma (IFN-γ), interleukin-1β (IL-1β), IL-2, IL-4, IL-5, IL-6, IL-7, IL-8, IL-10, IL-12p70, IL-13, IL-17A, macrophage inflammatory protein 1-beta (MIP-1β), monocyte chemoattractant protein-1 (MCP-1), tissue necrosis factor-alpha (TNF-α), and tissue necrosis factor-beta (TNF-β) in three study cohorts. Most cytokines were undetectable or extremely low in HC and CKD patients, while most ESKD patients had detectable or high levels. In particular, ESKD patients had signifcantly higher levels of IFN-γ, TNF-α, sCD40L, GM-CSF, IL-4, IL-8, MCP-1, and MIP-1β than CKD patients (Figure 1; Supplementary Figure 1). Altogether, these data confirm and extend previous evidence that kidney failure is associated with a pro-inflammatory state (15, 16).

### Naïve, Effector, and Memory CD4^+^ T Cells

**Figure 2:**
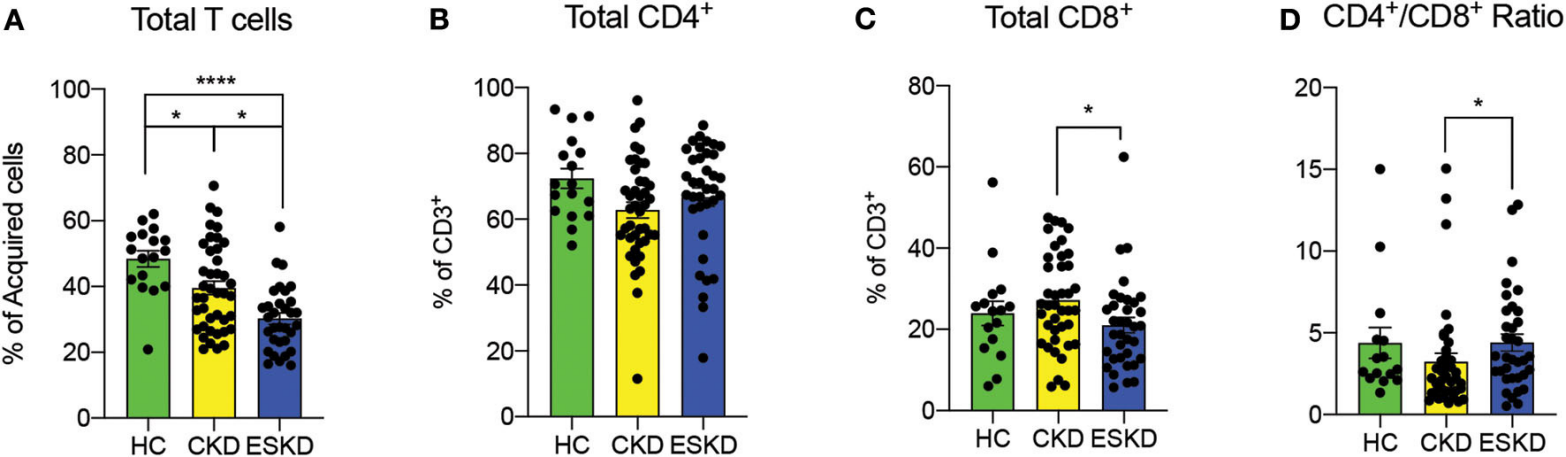
Total T cells, CD4^+^ and CD8^+^ cells and CD4^+^/CD8^+^ ratio in healthy control, CKD and ESKD patients. **(A)** CD3^+^ percentage of acquired cells; **(B,C)** CD4^+^ and CD8^+^ cells percentage of CD3^+^ T cells, and **(D)** CD4^+^/CD8^+^ ratio. Data are represented as mean and standard error of the mean (SEM). Each dot represents an individual value. *P < 0.05; ****P < 0.0001. Kruskal–Wallis test.

Consistent with prior reports (17–20), we observed fewer CD3^+^ T cells in CKD (39.5 ± 13.8%) and ESKD (30.3 ± 12.8%) when compared to HC (48.4 ± 10.1%) (Figure 2A). In total T cells, there was no difference in the percentage of total CD4^+^ T cells among the three groups. Between CKD and ESKD, ESKD group had a significantly lower percentage of total CD8^+^ T cells with a higher CD4^+^/CD8^+^ ratio (Figures 2B–D). Absolute numbers of CD4^+^ and CD8^+^ T cells of CKD and ESKD groups demonstrated similar findings to the percentage difference (Supplementary Figure 2), although we did not have absolute numbers in healthy controls during the collection process.

**Figure 3:**
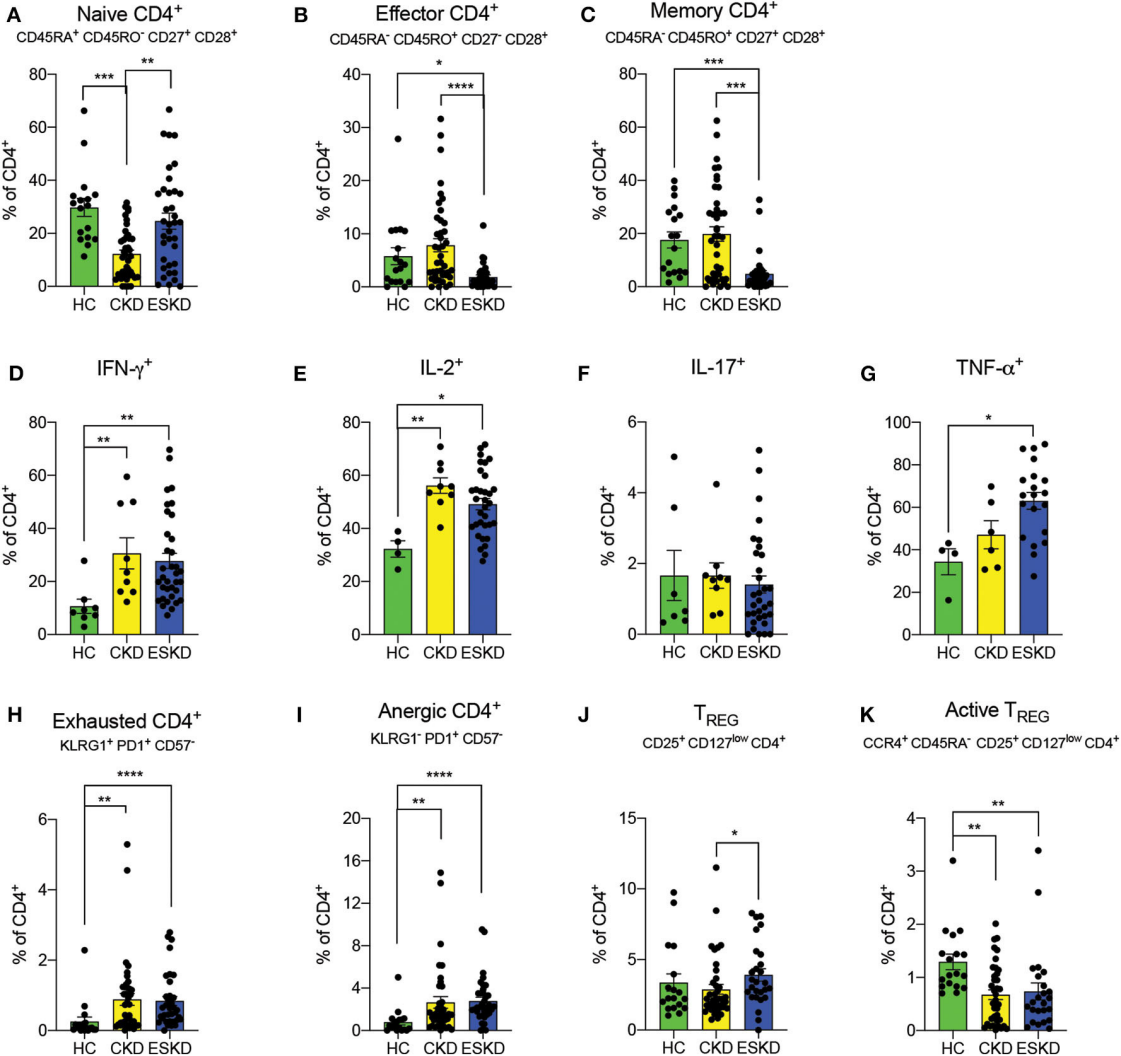
CD4^+^ T cell subsets in the three study groups. **(A–C)** Naïve, effector and memory CD4^+^ T cells. **(D–G)** IFN- γ, IL-2, IL-17, and TNF-α staining on CD4^+^ T cells. **(H,I)** Exhausted and anergic CD4^+^ T cells. **(J,K)** Total and active Treg. Data are represented as mean and standard error of the mean (SEM). Each dot represents an individual value. *P < 0.05; **P < 0.01; ***P < 0.001; ****P < 0.000.1. Kruskal–Wallis test. Gating strategies are shown in Supplementary Figures 3, 4.

CKD patients had the lowest frequency of naïve CD4^+^ T cells (CD45RA^+^CD45RO^−^CD27^+^CD28^+^), while ESKD patients had similar levels to HC (Figure 3A). Both frequencies of effector CD4^+^ T cells (CD45RA^−^CD45RO^+^CD27^−^CD28^−^) and memory CD4^+^ T cells (CD45RA^−^CD45RO^+^CD27^+^CD28^+^) were remarkably reduced in hemodialysis patients (Figures 3B,C). We next examined the intracellular levels of IFN-γ, IL-2, and TNF-α in CD4^+^ T cells, as a proxy of their function. We found that CD4^+^ T cells from CKD or ESKD patients demonstrated a more pro-inflammatory phenotype with increasing IFN-γ, IL-2, and TNF-α after mitogen stimulation, while IL-17 did not significantly differ across the three groups (Figures 3D–G).

### Dysfunctional and Regulatory CD4^+^ T Cells

Chronic inflammation has been associated with the emergence of T cell dysfunction (6). To test the hypothesis that a similar phenomenon occurs in patients with kidney failure, we measured phenotypically exhausted and anergic T cells. We found a significant increase of exhausted CD4^+^ (KLRG1^+^PD1^+^CD57^−^) and anergic T cells (KLRG1^−^PD1^+^CD57^−^) in both CKD and ESKD patients. (Figures 3H,I). Importantly, exhausted CD4+ T cells showed no cytokine production (Supplementary Figure 4).

Previous reports showed reduced levels of regulatory T cells (Treg) (21), a cell subset of CD4^+^ T cells essential for immune tolerance and eliminating chronic inflammation (22). Herein, we found a markedly decreased frequency of active Treg cells (CCR4^+^CD45RA^−^CD25^+^CD127^low−^CD4^+^CD8^−^CD3^+^) in both CKD and ESKD groups, whereas the percentage of total Treg (CD25^+^CD127^low^CD4^+^CD8^−^CD3^+^) in ESKD group was statistically higher than CKD but of no difference compared to HC (Figures 3J,K).

### Naïve, Memory, Effector, and Dysfunctional CD8^+^ T Cells

**Figure 4:**
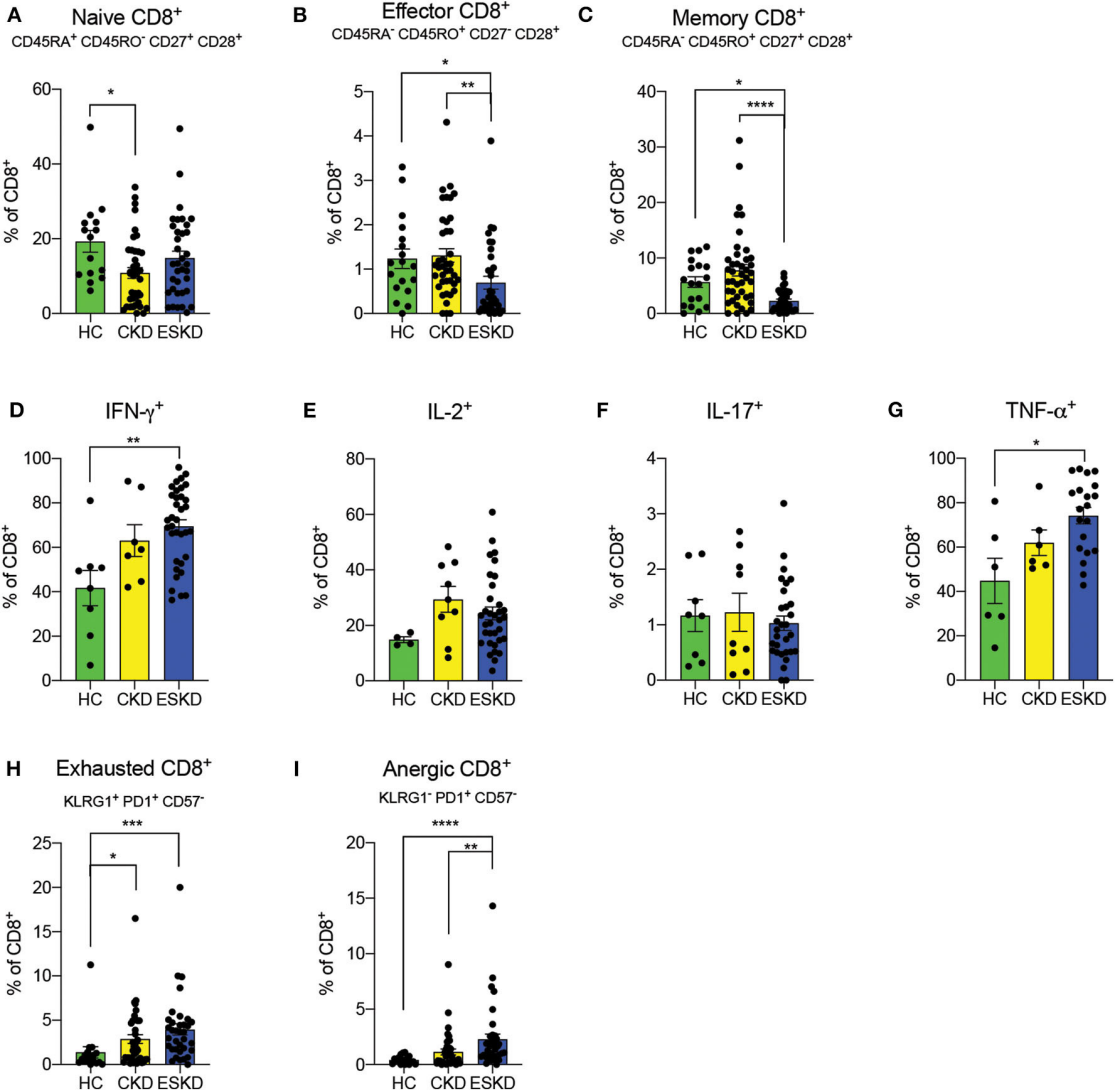
CD8^+^ T cell subsets in the three study groups. **(A–C)** Naïve, effector, and memory CD8^+^ T cells. **(D–G)** IFN-γ, IL-2, IL-17, and TNF-α staining on CD8^+^ T cells. **(H,I)** Exhausted and anergic CD8^+^ T cells. Data are represented as mean and standard error of the mean (SEM). Each dot represents an individual value. *P < 0.05; ****P < 0.000.1. Kruskal–Wallis test. Gating strategy is shown in Supplementary Figure 3.

Similar to CD4^+^ T cells, we observed a decreased frequency of naïve CD8^+^ T cells in CKD patients, while that of ESKD patients was close to HC (HC: 19.2 ± 11.1% vs. CKD: 10.8 ± 9.1% vs. ESKD: 14.83 ± 11.0%) (Figure 4A). ESKD patients had the lowest effector and memory CD8^+^ T cells among the groups (Figures 4B,C). In intracellular cytokine analyses, IFN-γ and TNF-α were significantly elevated after mitogen stimulation in ESKD. We also noticed a non-significant trend toward higher IL-2 levels in CKD and ESKD groups, but no difference in IL-17 across the groups (Figures 4D–G). Similar to CD4^+^ T cells, we found significantly more exhausted and anergic CD8^+^ T cells in ESKD patients (Figures 4H,I).

### Follicular Helper T Cells

**Figure 5:**
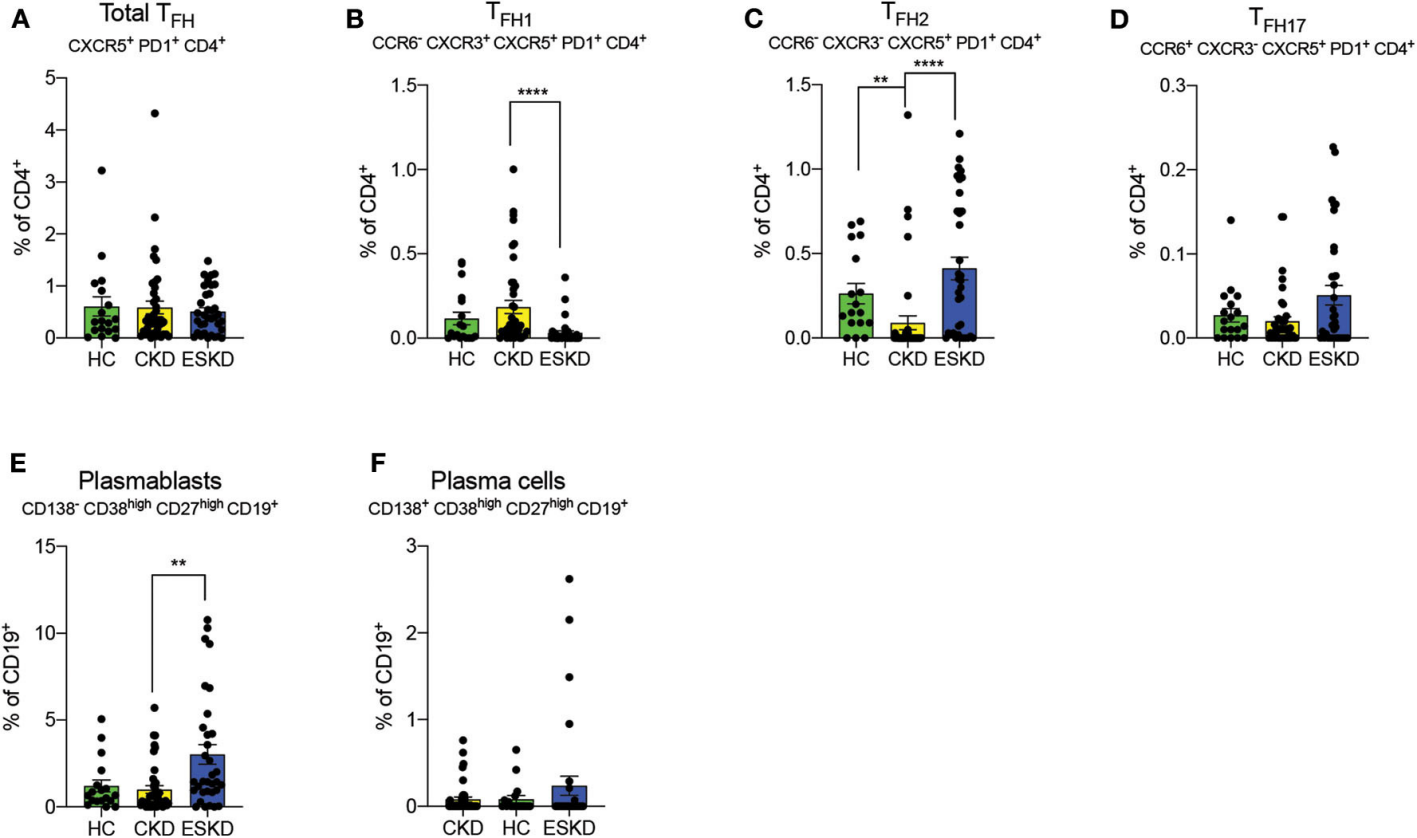
Follicular helper T cell subsets, plasmablasts, and plasma cells. **(A–D)** Total, T_FH1_, T_FH2_ and T_FH17_. **(E,F)** Plasmablasts and plasma cells on CD19^+^ B cells. Each dot represents an individual value. *P < 0.05; **P < 0.01; ****P < 0.000.1. Kruskal–Wallis test.

We did not observe differences in total T_FH_ (CXCR5^+^PD1^+^CD4^+^CD8^−^) amongst our cohorts (Figure 5A). ESKD patients had a remarkably lower frequency of T_FH1_ (CCR6^−^CXCR3^+^CXCR5^+^PD1^+^CD4^+^CD8^−^) cells but more T_FH2_ (CCR6^−^CXCR3^−^CXCR5^+^PD1^+^CD4^+^CD8^−^) and T_FH17_ cells (CCR6^+^CXCR3^−^CXCR5^+^PD1^+^CD4^+^CD8^−^) cells (albeit not significant; Figures 5B–D) among three cohorts. T_FH1_ has a pivotal role in response to viral infections (23, 24). By contrast, T_FH2_ have been suggested to provide help to B cells within GC, where they convert into plasma blasts and plasma cells and subsequently antibody secreting cells (25). Intriguingly, we also found a significant increase of circulating plasmablasts in ESKD group (Figure 5E) but not plasma cells (Figure 5F), suggestive of a partially promoted B cells maturation process.

## Discussion

Our study shows that kidney failure is associated with a pro-inflammatory state, characterized by increased T cell production of pro-inflammatory cytokines and high circulating levels of T_FH2_ and plasmablasts. These alterations may lead to a progressive dysfunction of immune cells, as supported by increased exhausted CD4^+^ and CD8^+^ T cells. Altogether, these findings were suggestive of persistent and active pro-inflammatory T cells in both CKD and ESKD patients.

Chronic inflammation can be triggered by reciprocal activation of innate and adaptive immunity (26). In our serum cytokine analysis, we identified a significant pro-inflammatory phenotype characterized by upregulated sCD40L, GM-CSF, IL-4, IL-8, MCP-1, and MIP-1β in the ESKD cohort, apart from the prototypical cytokines such as IFN-γ and TNF-α. CD40L is a costimulatory molecule predominantly expressed on activated CD4^+^ T cells. Activation of CD40-CD40L axis leads to activation of B cells and cytokine secretion, which in turn activate T cells as positive feedback (27). Increased sCD40L has been associated with chronic HIV infection (28), cancers (29), and pulmonary arterial hypertension (30), and could potentially serve as a biomarker of disease activities. Intriguingly, upregulated circulating sCD40L also enhanced expression and release of sCD40L in platelets (31), supporting the clinical observation of hypercoagulability in ESKD patients. IL-4 is produced by follicular helper T cells and has been shown to regulate B cell maturation in GC (32), which is consistent with our findings in upregulated T_FH2_ and increased plasmablasts. In addition, IL-4 (33) and MIP-1β (34) are known to polarize macrophage into M1 macrophage, a subtype of macrophage robustly producing cytokines and chemokines such as IL-1β and TNF-α. Although we did not include macrophage into our analysis, a prior review (35) has delineated the pro-inflammatory role of macrophages in kidney failure patients. Of note, the conventional intermittent hemodialysis membranes do not remove middle molecules (500 Da-15 kDa), and therefore the higher levels of pro-inflammatory cytokines were not due to poor clearance compared with others of no statistical significance. Thus, our results support a chronically activated inflammatory state in ESKD.

Regarding T cells, first we noted a similar finding of worsening lymphopenia in patients with CKD and ESKD. However, we did not observe a decreased or inverted ratio of CD4^+^/CD8^+^ T cells as previously reported by others (11, 19). This discrepancy might be explained by the unknown status of CMV infection of our patients, as Crépin et al. (18) reported that inverted ratio was only found in CMV-seropositive patients in their cohort. Lymphopenia in CKD/ESKD patients, especially naïve CD4^+^ and CD8^+^ T cells and T central memory, was previously attributed to decreased thymic output, increased apoptosis or shortening of telomere length (36). We also observed reduced naïve CD4^+^ and CD8^+^ T cells in CKD but not in ESKD cohort. Of note, we strictly defined naïve T cells as CD45RA^+^CD45RO^−^CD27^+^CD28^+^ in our study rather than CD45RO^−^CCR7^+^, which might represent a different population when compared to others (37). It is unclear whether the mobilization of naïve T cells in our ESKD group was mediated by intermittent removal of uremic toxins by hemodialysis or other factors.

Our data demonstrate an association between ESKD and reduced effector CD4^+^ and CD8^+^ T cells. Whether this reduction is due to recruitment of these cells to the peripheral tissues or reflects an absolute decline goes beyond the scope of the current study. However, after mitogen stimulation, T cells from ESKD patients demonstrated a pro-inflammatory phenotype with an increase in IFN-γ, IL-2 and TNF-α, whereas CKD patients only had a higher level of IL-2. Altogether, these findings were suggestive of persistent and active pro-inflammatory T cells especially in ESKD patients.

Intriguingly, we found that both CKD and ESKD patients had higher frequencies of exhausted and anergic CD4^+^ and CD8^+^ T cells. T cell receptor (TCR) signaling is essential for T cell differentiation into effector cells as well as appropriate clonal expansion (38) and defective TCR signaling have been described in elderly individuals (39) and chronic inflammatory diseases (40). In our study, phenotypically exhausted T cells were not capable of effectively producing cytokines. Therefore, it is tempting to speculate that, upon chronic stimulation (possibly driven by uremic toxins, amongst other stimuli), T cells become dysfunctional, leading to increased risk of infections and malignancies (1).

Despite the inconsistent results of Treg frequency in ESKD patients when compared to HC (17, 21, 41, 42), impaired *ex vivo* Treg proliferation and suppressive capacity has been shown in patients with ESKD (21). Treg counteracts effector T cells to promote self-tolerance and reduce alloimmunogenicity. Forkhead box P3 (FOXP3), a pivotal transcription factor in Treg homeostasis, is degraded in chronic inflammation by pro-inflammatory cytokines such as TNF-α (43). Thus, downregulated Treg can result in excessive inflammatory responses and aberrant autoimmune diseases. In our cohort, we observed a slight increase in the total frequency of Treg in ESKD patients but a significant decrease of active Treg (CCR4^+^CD45RA^−^CD25^+^CD127^low−^CD4^+^CD8^−^CD3^+^) in both CKD and ESKD patients. This supports the hypothesis that the chronic inflammatory milieu in ESKD impairs Treg function, instead of affecting their number. A similar finding of upregulation of total Treg was considered to be a reactive response in a cohort ANCA-associated vasculitis (AAV) patients (44).

Upregulation of T_FH_ cells has been associated with chronic inflammation in cardiovascular (45, 46) and autoimmune diseases (47). In our cohort, we observed T_FH1_, a possibly protective subtype against viral infection, clearly diminished in ESKD. On the contrary, the presence of pro-inflammatory T_FH2_ and T_FH17_ was associated with systemic lupus erythematous (SLE) (48) and rheumatoid arthritis (49), respectively, and T_FH2_ was remarkably increased in our ESKD cohort. To date, there was only one small cohort of ESKD patients, where Assing et al. (50) reported a decreased frequency of peripheral T_FH_ from apoptosis. The authors also suggested the reduced T_FH_ could explain antibody deficiency in ESKD from a disturbed interaction between T_FH_ and B cells in GC. However, the authors did not further classify the subtypes of T_FH_ in their analysis. Indeed, B cells lymphopenia has been shown in patients with CKD or ESKD due to B cell apoptosis and poor B cell differentiation (51, 52), but the mechanism remains poorly understood especially in the context of T_FH_ and B cell interactions. In line with T_FH_ findings, we also observed a higher percentage of circulating plasmablasts, suggesting an increase of B cell maturation within GC. Surprisingly, the upregulated plasmablasts did not result in a similar increase of plasma cells in ESKD patients. After entering circulation from secondary lymphoid tissue, plasmablasts further differentiate into plasma cells, the predominant antibody-secreting cells, and later transition into long-lived plasma cells. This process requires necessary survival factors and environment (bone marrow or spleen) to maintain circulating plasma cells in the periphery (53). For instance, deletion of B cell maturation antigen (BCMA), a B-cell activating factor (BAFF) receptor, was shown to affect the survival of long-lived plasma cells in a murine model (54). Downregulated BAFF receptor on transitional B cells has been implicated in deficient B cell differentiation in ESKD patients (51) but whether it also occurs in plasma cells requires further studies.

Our study has certain limitations. The numbers of our patients were relatively small and the cross-sectional nature of the study prevents testing the association between immunological abnormalities and cardiovascular outcomes, incidence of malignancies, or infectious complications. We did not have information of CMV infection status of our cohort, which could partly contribute to T cell exhaustion. In conclusion, we showed that kidney failure is associated with a pro-inflammatory state that may be responsible for the exhausted phenotype of T cells and the skewed upregulation of T_FH2_, especially in ESKD. These immunological changes may be, at least in part, responsible for their increased cardiovascular risk and the augmented susceptibility to infections and malignancies.

## Data Availability Statement

The raw data supporting the conclusions of this article will be made available by the authors, without undue reservation.

## Ethics Statement

The studies involving human participants were reviewed and approved by IRB-20-03454, Mount Sinai Hospital. The patients/participants provided their written informed consent to participate in this study.

## Author Contributions

PC designed the study. SH, SB, CC, and MH performed all the experiments. JM, AA, BM, WZ, and LG provided patient samples. SH, JL, SM-WY, GLM, and PC helped with data interpretation and wrote the initial draft of the paper. All authors approved the paper.

## Conflict of Interest

The authors declare that the research was conducted in the absence of any commercial or financial relationships that could be construed as a potential conflict of interest.
