# Supplementary material for: Kidney Failure Associates With T Cell Exhaustion and Imbalanced Follicular Helper T Cells
Source: Front Immunol. 2020 Sep 29;11:583702. doi: 10.3389/fimmu.2020.583702 (PMC7552886; doi:10.3389/fimmu.2020.583702)
Supplement: Supplementary file 1 [file Table_1.pdf]

Supplementary Table 1. List of cell subsets analyzed by flow cytometry.

Subset name	Markers	Parent Population
Naïve CD4 T cells	CD8-CD4+CD45RA+CD45RO-CD28+CD27+	Singlets
Effector CD4 T cells	CD8-CD4+CD45RA-CD45RO+CD28+CD27-	Singlets
Memory CD4 T cells	CD8-CD4+CD45RA-CD45RO+CD28+CD27+	Singlets
Naïve CD8 T cells	CD4-CD8+CD45RA+CD45RO-CD28+CD27+	CD4-CD8+
Effector CD8 T cells	CD4-CD8+CD45RA-CD45RO+CD28+CD27+	Singlets
Memory CD8 T cells	CD4-CD8+CD45RA-CD45RO+CD28+CD27-	Singlets
TREG	CD3+CD8-CD4+CD25+CD127low	CD3+CD8-CD4+
Active TREG_1	CD3+CD8-CD4+CD25+CD127lowCCR4+CD45RA-	CD3+CD8-CD4+
Active TREG_2	CD3+CD8-CD4+CCR4+CD45RA-CD25+CD127low	CD3+CD8-CD4+CCR4+CD45RA-
Total T cells	CD3+	Acquired cells
CD4 T cells	CD3+CD8-CD4+	CD3+
Exhausted CD4 T cells_1	CD3+CD8-CD4+PD1+CD57-	CD3+CD8-CD4+
Exhausted CD4 T cells_2	CD3+CD8-CD4+PD1+CD57-KLRG1+	CD3+CD8-CD4+PD1+CD57-
Anergic CD4 T cells_1	CD3+CD8-CD4+PD1+CD57-KLRG1-	CD3+CD8-CD4+PD1+CD57-
Senescent CD4 T cells_1	CD3+CD8-CD4+PD1-CD57+KLRG1+	CD3+CD8-CD4+PD1-CD57+
Senescent CD4 T cells_2	CD3+CD8-CD4+CD57+CCR7+	CD3+CD8-CD4+
Senescent CD4 T cells_3	CD3+CD8-CD4+CD28-	CD3+CD8-CD4+
Total PD1 CD4	CD3+CD8-CD4+PD1+	CD3+CD8-CD4+
CD8 T cells	CD3+CD4-CD8+	CD3+
Exhausted CD8 T cells_1	CD3+CD4-CD8+PD1+CD57-	CD3+CD4-CD8+
Exhausted CD8 T cells_2	CD3+CD4-CD8+PD1+CD57-KLRG1+	CD3+CD4-CD8+PD1+CD57-
Anergic CD8 T cells_1	CD3+CD4-CD8+PD1+CD57-KLRG1-	CD3+CD4-CD8+PD1+CD57-
Senescent CD8 T cells_1	CD3+CD4-CD8+PD1+CD57+	CD3+CD4-CD8+PD1-CD57+
Senescent CD8 T cells_2	CD3+CD4-CD8+CD57+CCR7+	CD3+CD4-CD8+
Senescent CD8 T cells_3	CD3+CD4-CD8+CD28-	CD3+CD4-CD8+
Exhausted CD4 T cells_3	CD3+CD8-CD4+PD1+CD57-KLRG+	CD3+CD8-CD4+
Anergic CD4 T cells_2	CD3+CD8-CD4+PD1+CD57-KLRG1-	CD3+CD8-CD4+
Senescent CD4 T cells_2	CD3+CD8-CD4+PD1-CD57+KLRG1+	CD3+CD8-CD4+
Exhausted CD8 T cells_3	CD3+CD4-CD8+PD1+CD57-KLRG1+	CD3+CD4-CD8+
Anergic CD8 T cells_2	CD3+CD4-CD8+PD1+CD57-KLRG1-	CD3+CD4-CD8+
Senescent CD8 T cells_2	CD3+CD4-CD8+PD1-CD57+KLRG1+	CD3+CD4-CD8+
Total PD1 CD8	CD3+CD4-CD8+PD1+	CD3+CD4-CD8+
Total TFH cells	CD8-CD4+CXCR5+PD1+	CD8-CD4+
TFH17	CD8-CD4+CXCR5+PD1+CCR6+CXCR3-	CD8-CD4+
TFH1	CD8-CD4+CXCR5+PD1+CCR6-CXCR3+	CD8-CD4+
TFH2	CD8-CD4+CXCR5+ PD1+CCR6-CXCR3-	CD8-CD4+
Plasmablasts	CD3-CD56-CD19+CD38highCD27highCD138-	CD3-CD56-CD19+
Plasma cells	CD3-CD56-CD19+CD38highCD27highCD138+	CD3-CD56-CD19+
IFN-γ+ CD4 T cells	CD8-CD4+IFN-γ+	CD8-CD4+
IL2+ CD4 T cells	CD8-CD4+IL2+	CD8-CD4+

IL17+ CD4 T cells	CD8-CD4+IL17+	CD8-CD4+
TNF-α+ CD4 T cells	CD8-CD4+TNF-α+	CD8-CD4+
IFN-γ+ PD1- CD4 T cells	CD8-CD4+PD1-IFN-γ+	CD8-CD4+
IL2+ PD1- CD4 T cells	CD8-CD4+PD1-IL2+	CD8-CD4+
IL17+ PD1- CD4 T cells	CD8-CD4+PD1-IL17+	CD8-CD4+
TNF-α+ PD1- CD4 T cells	CD8-CD4+PD1-TNF-α+	CD8-CD4+
IFN-γ+ PD1-+CD4 T cells	CD8-CD4+PD1+IFN-γ+	CD8-CD4+
IL2+ PD1+ CD4 T cells	CD8-CD4+PD1+IL2+	CD8-CD4+
IL17+ PD1+ CD4 T cells	CD8-CD4+PD1+IL17+	CD8-CD4+
TNF-α+ PD1+ CD4 T cells	CD8-CD4+PD1+TNF-α+	CD8-CD4+
IFN-γ+ CD8 T cells	CD4-CD8+IFN-γ+	CD4-CD8+
IL2+ CD8 T cells	CD4-CD8+IL2+	CD4-CD8+
IL17+ CD8 T cells	CD4-CD8+IL17+	CD4-CD8+
TNF-α+ CD8 T cells	CD4-CD8+TNF-α+	CD4-CD8+
IFN-γ+ PD1- CD8 T cells	CD4-CD8+PD1-IFN-γ+	CD4-CD8+
IL2+ PD1- CD8 T cells	CD4-CD8+PD1-IL2+	CD4-CD8+
IL17+ PD1- CD8 T cells	CD4-CD8+PD1-IL17+	CD4-CD8+
TNF-α+ PD1- CD8 T cells	CD4-CD8+PD1-TNF-α+	CD4-CD8+
IFN-γ+ PD1+ CD8 T cells	CD4-CD8+PD1+IFN-γ+	CD4-CD8+
IL2+ PD1+ CD8 T cells	CD4-CD8+PD1+IL2+	CD4-CD8+
IL17+ PD1+ CD8 T cells	CD4-CD8+PD1+IL17+	CD4-CD8+
TNF-α+ PD1+ CD8 T cells	CD4-CD8+PD1+TNF-α+	CD4-CD8+
