# Supplementary figures and images for: Kidney Failure Associates With T Cell Exhaustion and Imbalanced Follicular Helper T Cells
Source: Front Immunol. 2020 Sep 29;11:583702. doi: 10.3389/fimmu.2020.583702 (PMC7552886; doi:10.3389/fimmu.2020.583702)

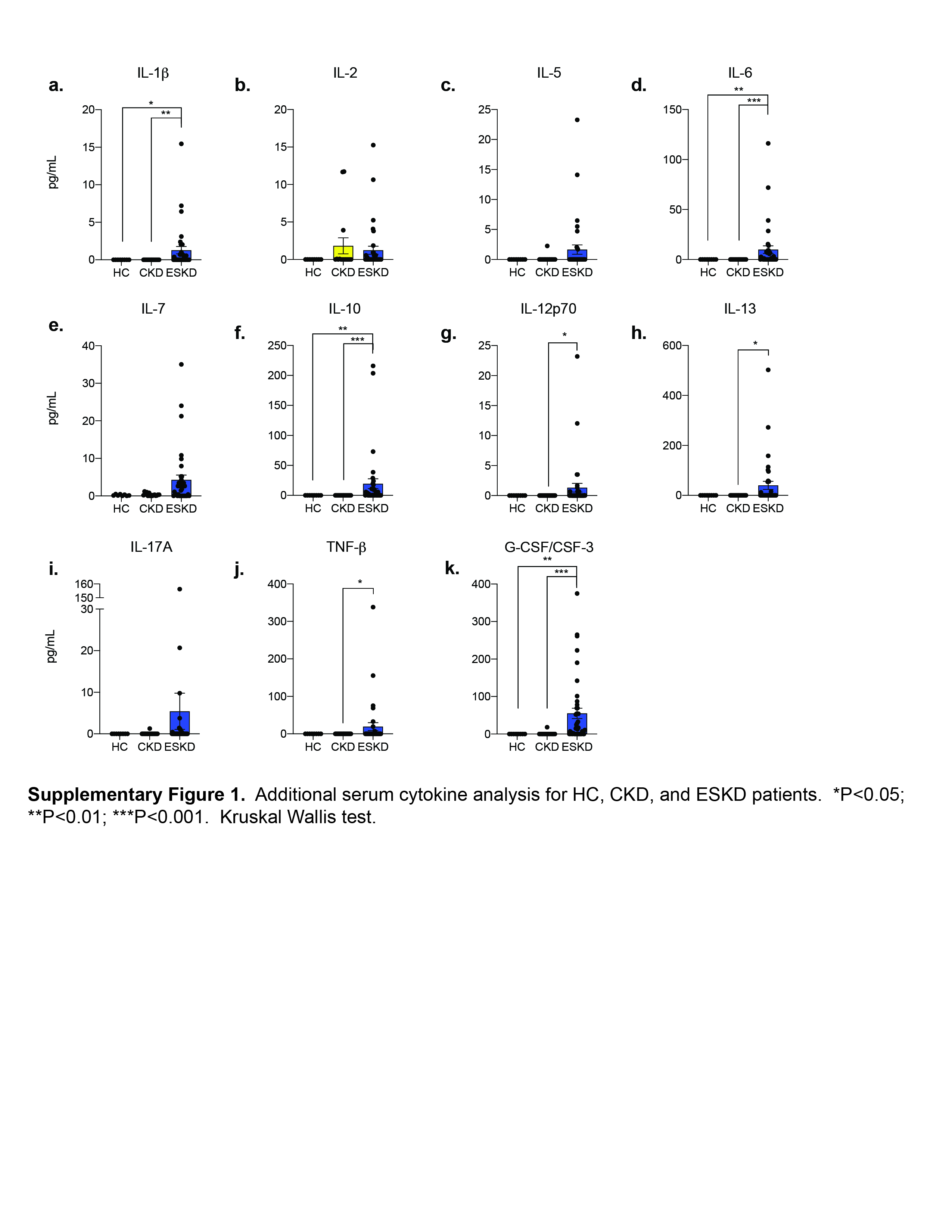

Supplement: Supplementary file 2 [file Image_1.TIF]

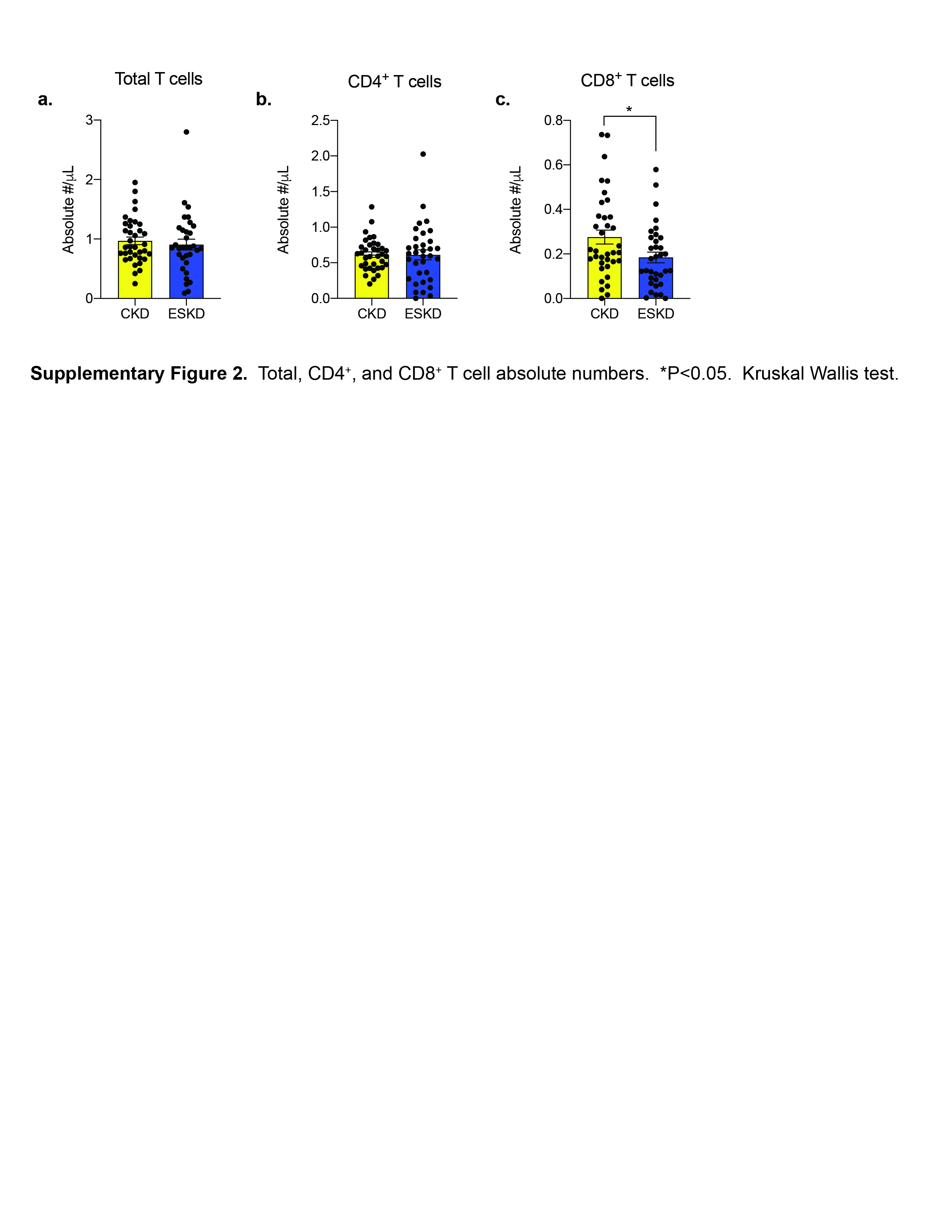

Supplement: Supplementary file 3 [file Image_2.TIF]

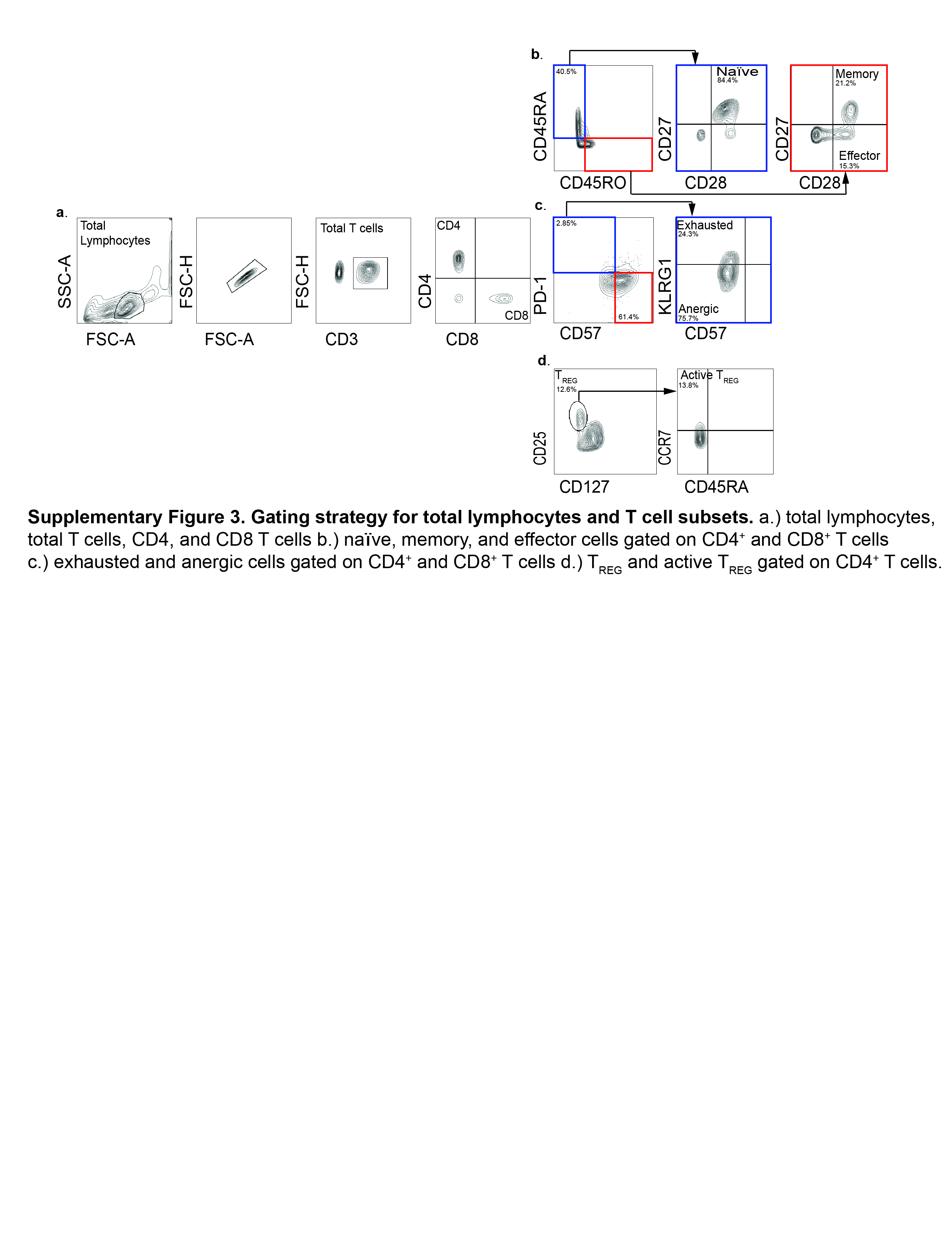

Supplement: Supplementary file 4 [file Image_3.TIF]

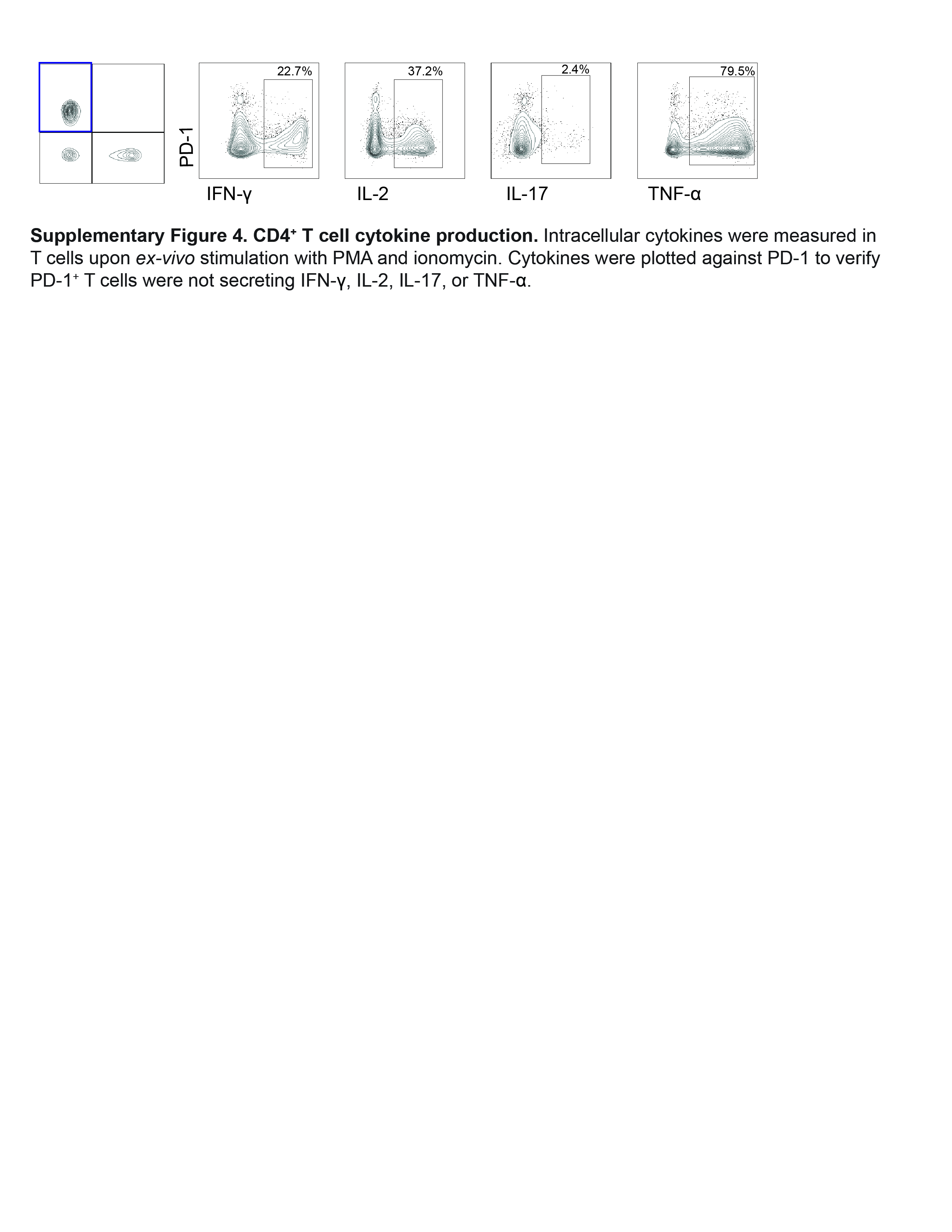

Supplement: Supplementary file 5 [file Image_4.TIF]
